# Genotypic characterization of extended spectrum beta-lactamase in gram negative bacterial contaminants of some door handles in Olabisi Onabanjo University Teaching Hospital, Sagamu, Ogun state

**DOI:** 10.4314/ahs.v23i2.23

**Published:** 2023-06

**Authors:** Oluwatoyin Bola Famojuro, Ifeoluwa Omolola Adesanya, Jerome Olu Ajewole, Tayo Ibukun Famojuro

**Affiliations:** 1 Department of Pharmaceutical Microbiology, Faculty of Pharmacy, Olabisi Onabanjo University, Sagamu, Ogun state; 2 Department of Medical Microbiology and Parasitology, Olabisi Onabanjo University Teaching Hospital, Sagamu, Ogun state; 3 Department of Pharmacognosy, Faculty of Pharmaceutical Sciences, Bingham University, Karu Nasarawa state

**Keywords:** Bacterial contamination, drug resistance, extended spectrum beta-lactamase

## Abstract

**Background:**

Nosocomial infections due to cross-transmission by microorganisms from inanimate surfaces have become a recognized public health problem.

**Objectives:**

This research was carried out to detect bacterial contaminants of door handles and their production of extended spectrum beta-lactamase (ESBL) in Olabisi Onabanjo University Teaching Hospital.

**Methods:**

Seventy door handles from twenty-seven sections of Olabisi Onabanjo University Teaching Hospital were swabbed and inoculated on different culture media. The isolates were identified using standard microbiological procedures. Susceptibility of the isolates to eleven antibiotics was determined by Kirby- Bauer disc diffusion test. Phenotypic detection of ESBL was determined by modified double disc synergy test (MDDST) followed by PCR amplification of ESBL- encoding genes.

**Results:**

Forty-four (62.9%) out of 70 door swabs were positive for bacterial growth. *S . aureus* was the most common isolate (22.9%). Multidrug resistance was observed in 15(79.0%) and 20(69%) of Gram positive and Gram-negative isolates, respectively. Coagulase negative staphylococci, *E. coli, Acinetobacter* and *Shigella* species were totally resistant to ceftazidime (100%). PCR amplification of ESBL-genes revealed the presence of blaTEM blaCTX -M and blaSHV in 4(14.3%), 2(7.2%) and 1(3.6%) isolate, respectively.

**Conclusion:**

This study revealed that door handles of different sections in hospital environment were contaminated by multidrug resistant Gram-negative bacteria encoding ESBL genes.

## Introduction

Hospital acquired infections (HAIs) constitute a significant public-health issue due to their high recurrence rate, severity, economic and social costs. According to the WHO, more than 1.4 million individuals around the world are considered to be suffering from infectious complications acquired in hospitals at any given point in time 1. The increased burden of hospital acquired infections in developing countries disproportionately affects high-risk populations, such as ICU patients and newborns, with HAI rates several times higher than in advanced countries 2 3. In settings with resource constraints, HAI incident rates vary considerably between 0.4 and 9.2%, 3. The problem is probably worse, however, the extent of the problem is still underestimated or unknown, given the difficulties of HAI diagnosis and the lack of surveillance activities in most of these countries, which necessitate resources and expertise. Moreover, many healthcare facilities lack effective infection control systems and skilled personnel, resulting in primitive infection control measures 2 4.

Many pathogenic microorganisms have been discovered on hospital surfaces such as chairs, tables, floors, door handles, stethoscopes, bathroom floors, bed linen, sinks, tables, keyboards, ultrasound transducers and office equipment 5,6,7,8. Various researchers have demonstrated the possible role of the environment as a reservoir for patient contamination due to the presence of multidrug-resistant bacteria 9,10. Many bacteria can survive in animate surfaces for weeks in the hospital 11,12. Bacteria can form biofilms on the surface, which serves as a breeding ground for them. Temperature, humidity, the influence of organic matter and prevalence of infection control practices, all also play a role in the colonization and survival of pathogens 13.

Door handles have been documented as pathogen breeding places and as the main focus of high-risk common touch surfaces that enhance bacterial spread within hospital wards. Poor hand hygiene has been associated with the spread of nosocomial pathogens. Touching the environment of patients is more likely to contaminate healthcare workers' hands than touching patients themselves 14. Hand hygiene has been identified as one of the most efficient and significant ways to prevent pathogens connected with hospital environment 1.

Infection prevention and control in health care facilities necessitate the monitoring and assessment of hospital door handles because contact with door handles contaminated by people who do not practice hand hygiene may increase the risk of infection 15. Gloves and other cross-contaminated items found in the hospital surroundings can also contaminate door handles 16. Considering the possible risk that pathogen-infested hospital door handles pose to users, as well as the problems associated with ESBL-producing Gram-negative bacteria, this study aimed to identify bacterial contaminants, determine the level of bacterial contamination, and identify extended-spectrum beta-lactamase-producing Gram-negative bacteria on door handles of the Olabisi Onabanjo University Teaching Hospital building.

## Materials and Methods

### Ethical approval

This study was approved by the Health Research Ethics Committee of Olabisi Onabanjo University Teaching Hospital, Sagamu, Ogun state before the study commenced.

### Study area

Maternity ward, Laboratory, Radiology, Main record, Female surgical ward, Male medical ward, Children ward, Female medical ward, Audit, Virology, Accident and Emergency, Nursing room, Nursing station, Medical social service, Dialysis centre, General outpatient, In -patient, Blood bank, Phlebotomy, Children emergency, Chief nursing officer Eye clinic, Paediatric outpatient clinic, Consulting room, Modular theatre, Pharmacy and Tailoring Departments of Olabisi Onabanjo Teaching Hospital, Sagamu, Ogun state.

### Collection of samples

A total of 70 samples were collected by rubbing and rotating sterile swabs moistened with normal saline on door handles (approximately 1.5 cm) of different sections/ departments of Olabisi Onabanjo University Teaching Hospital, Sagamu. The swabs were transported immediately to the laboratory.

### Isolation and identification of bacterial isolates

Samples were inoculated on blood agar and MacConkey agar, and incubated at 37°C for 24 hours. The bacterial colonies obtained were subculture into nutrient broth, and following incubation for 24hrs at 37°C, the broth cultures were further streaked on eosin methylene blue agar, Cetrimide agar, mannitol salt agar, MacConkey agar and salmonella-shigella agar plates. Standard microbiological techniques such as colony morphology, microscopic characteristics and conventional biochemical tests such as citrate, methyl red, Voges-Proskauer, oxidase, catalase, indole, urease, hemolysis, gelatin hydrolysis, gas production, motility, hydrogen sulphide production and coagulase tests as well as sugar fermentation on triple sugar iron (TSI) agar were used to identify the isolates 17. Distinct colonies were inoculated on agar slants and refrigerated until further use.

### Antibiotic susceptibility test

The Kirby Bauer disc diffusion method was used. The isolates were tested for susceptibility to standard antibiotic discs; Levofloxacin (5µg), Imipenem (10µg), Azithromycin (15µg), Cefuroxime (30µg), Gentamicin (10µg), Carbenicillin (30µg), Ceftazidime (30µg), cefepime (30µg), cefotaxime (30µg), Cephalexin (30µg), Augmentin (amoxicillin/clavulanic acid (20µg/10µg)). The isolates were sub-cultured on nutrient agar plate and incubated for 24 hours. Then, 3-4 colonies of each isolate were picked using a sterile inoculating loop and suspended into sterile distilled water to give turbidity equivalent to McFarland standard of density 1.5 x 10^8^ CFU/mL. Molten Mueller Hinton Agar (MHA) was poured into a Petri dish and allowed to solidify. The surface of the Mueller Hinton agar was inoculated with the test organism using sterile swab stick. The discs were placed on the agar plates using a sterile pair of forceps and allowed to stand for 30 minutes. The inoculated agar plates were incubated in an inverted position for 24 hours at 37°C. The zones of growth inhibition were measured to the nearest millimetres and interpreted as sensitive, intermediate and resistance using the performance standards for antimicrobial susceptibility testing 18.

### Phenotypic detection of extended spectrum beta lactamase (ESBL)

The isolates from door handles were tested for extended spectrum beta lactamase using modified double disc synergy test (MDDST). The bacterial colonies obtained were subculture into nutrient broth, and following incubation for 24hrs at 37°C, the broth cultures were further streaked on nutrient agar, then 3- 4 colonies of each isolate was picked and suspended in sterile distilled water, and was serially diluted to give turbidity equivalent to McFarland standard (1.5 x 10^8^ CFU/mL). Then, 20ml of Mueller-Hinton agar after cooling to 45-50^o^C was poured into 90mm diameter sterile Petri dish to depth of 4mm. The plates were dried for immediate use for 10-30 minutes at 36oC by placing them in an upright position in a dry heat oven. The Mueller-Hinton agar plates were streaked with the isolate's suspension using a sterile swab stick. Then, Amoxicillin-clavulanic acid (30µg) was placed at the center of the plate, and disc containing Cefotaxime (30µg), Cefepime (30µg) and Ceftazidime (30µg) were placed 2cm (center to center) from Amoxicillin-clavulanic acid disc with the aid of a sterile pair of forceps. The plates were then incubated at 37oC for 24 hours, a clear extension of the edge of the zone of growth inhibition of cephalosporins towards Amoxicillin-clavulanic acid disc was interpreted positive for ESβL production 18.

### Genotypic detection of ESBLs in Gram-negative isolates

#### Extraction of DNA

The bacterial chromosomal DNA was extracted by a boiling method. Briefly, 18-24 h from tryptic soy agar (TSA) was inoculated in 2 ml Luria Bertani broth (LB) and incubated for18-24 h. LB broth were centrifuged (10 000 RPM/min for 10 min) and bacterial cells were suspended in 500 µl of phosphate buffer (100 mM, pH 7) to weaken the membranes and immersed in a boiling water bath at 100°C for 15 min to release the genetic material. The DNA was then precipitated with 250 µL of absolute alcohol and washed twice in 1000 µL of 70% alcohol. The DNA was then re-suspended in 100 µL of sterile water 19.

#### PCR amplification of ESBL genes in Gram negative isolates

The ESBL genes, blaTEM, blaSHV and blaCTX-M were detected by PCR using the thermal cycler (Applied Biosystems, USA). The sequences of the different primers are presented in Table 3.1. Two PCR cycles were performed: one duplex for blaTEM and blaSHV, and one simplex for blaCTX-M 20, 21.

**Table 1:** Primer sequences of ESBL genes

ESBL genes	Sequence (5′-3′)	Amplicon size (bp)	References
CTX-M-9	For: TTTGCGATGTGCAGTACCAGTAA Rev: CGATATCGTTGGTGGTGCCATA	544	Edelstein et al. [19]
TEM	For: GAGTATTCAACATTTTCGT Rev: ACCAATGCTTAATCAGTGA	857	Maynard et al. 20
SHV	For: TCGCCTGTGTATTATCTCCC Rev: CGCAGATAAATCACCACAATG	768	Maynard et al. 20

The PCR final volume of 25 µL consisted of 1 µL of DNA, 12.5 µl of WizPure™ PCR 2X Master (Wizbiosolutions, South Korea), 1 µL of each primer (0.2 pmol/µl) and 9.5 µL molecular grade water. The amplification condition for blaTEM, blaSHV and blaCTX-M consist of initial denaturation 94°C for 10 min. 30 cycles of denaturation at 94°C for 40 s, annealing at 60°C for 40 s and elongation at 72°C for 1 min with a final elongation step at 72°C for 7 min. To visualize PCR products, a 2 percent agarose gel stained with 0.5 µg/ml of ethidium bromide was electrophoretically migrated at 100 volts for 1 hour. A marker of 100 bp was used as reference. After migration, the various bands were observed under UV illumination.

### Statistical analyses

The difference between the departments and the type of bacteria identified was determined using Pearson Chi square test. Statistical significance was taken as p<0.05.

## Results

### Isolation and identification of isolates

**Figure 1:**
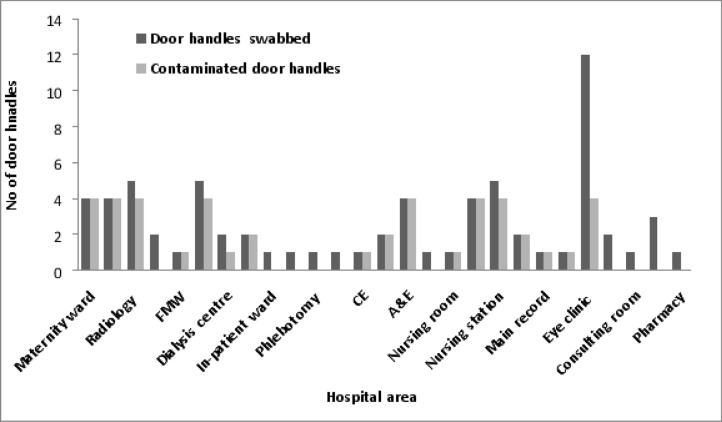
Frequency distributions of hospital door handles MSS= Medical social service; CNO= Chief nursing officer; FMW= Female medical ward; FSW= Female surgical ward; MMW= Male medical ward; CE= Children emergency; A&E = Accident and emergency; POC= Paediatric outpatient clinic; TD= Tailoring department

A total of 70 samples were collected from twenty-seven (27) sections of Olabisi Onabanjo University Teaching Hospital Sagamu by swabbing, with 44 samples yielding 48 bacterial isolates. Out of 27 sections, 17(63.0%) were positive for bacterial culture. No bacteria were isolated from door swabs of ten sections including Audit, In-patient, Blood bank, Phlebotomy, Children ward, Paediatric outpatient clinic, Consulting room, Modular theatre, Pharmacy and Tailoring sections. Forty-four (62.9%) out of 70 door swabs were positive for bacterial growth (Figure 1). The bacterial species found were *Staphylococcus aureus, Salmonella* species, coagulase negative staphylococci, *Klebsiella pneumoniae, Pseudomonas aeruginosa, Shigella* species, *Acinetobacter* species and *Escherichia coli*. A total of forty-eight (48) isolates were identified. *Staphylococus aureus* was the most common isolate 11(22.9%), followed by coagulase negative staphylococci 8(16.7%) and *K pneumoniae* 8(16.7%) while *Acinetobacter* and *Shigella* species were the least (4.2%) (Table 2).

**Table 2:** Frequency of isolated bacterial species

Isolated bacterial species	No of isolated bacteria	% Of isolated bacteria
*Staphylococcus aureus*	11	22.9
Coagulase negative staphylococci (CoNS)	8	16.7
*Escherichia coli*	6	12.5
*Klebsiella pneumonia*	8	16.7
*Acinetobacter baumannii*	2	4.2
*Pseudomonas aeruginosa*	7	14.6
*Salmonella* species	4	8.3
*Shigella* species	2	4.2
**Total**	**48**	**100**

Table 3 shows the number of isolates and the types of bacterial species found on door handles in relation to different sections of the hospital. *P. aeruginosa* and *Shigella* species were the most predominant isolate from the Laboratory Department while *E. coli* was the most common isolate from main medical ward. Pearson chi square was used to determine whether there was a relationship between the department and the bacteria isolated. P value was >0.05, hence, no significance on the type of bacteria identified and the department.

**Table 3:** Isolates found on door handles in relation to different sections of the hospital

Hospital area	*S. aureus*	CoNS	*P. aeruginosa*	*K. pneumoniae*	*E. coli*	*Acinetobacter* species	*Shigella* species	*Salmonella* species	Total
Maternity ward	2	2	0	1	0	0	0	1	**6**
Laboratory	1	0	4	2	0	0	1	3	**11**
Radiology	1	1	0	0	0	0	0	0	**2**
Audit		0	1	0	0	0	0	0	**1**
FMW	1	0	0	1	0	0	0	0	**2**
Virology	1	1	0	1	0	0	0	0	**3**
Accident and emergency	1	0	0	0	0	0	1	0	**2**
Nursing room	1	0	0	0	0	0	0	0	**1**
Male medical ward	1	1	0	1	3	0	0	0	**6**
Nursing station	0	2	1	0	0	1	0	0	**4**
Female surgical ward	1	0	0	1	1	0	0	0	**3**
Main record	0	1	0	0	0	0	0	0	**1**
Chief Nursing Officer	1	0	0	1	0	0	0	0	**2**
Eye clinic	0	0	1	0	2	1	0	0	**4**
**Total**	**11**	**8**	**7**	**8**	**6**	**2**	**2**	**4**	**48**

### Antibiotic susceptibility profile of the isolates

All CoNS, *Acinetobacter, Salmonella*, and *Shigella* species were susceptible to imipenem. *Acinetobacter* species was the most resistant isolates against augmentin (100%), followed by *K. pneumoniae* (87.5%). Coagulase negative staphylococci, *Acinetobacter* species and Shigella species were totally resistant to ceftazidime (100%). *E. coli* and *Acinetobacter* species were the most resistant to cefepime and cefotaxime (Table 4). Among the Gram-positive isolates, 15(79.0%) were multidrug resistant while 20(69%) of Gram-negative isolates were multi drug resistant. In Gram positive isolates, *S. aureus* (90.9%) showed the highest multidrug resistance while coagulase negative staphylococci (CoNS) had 63.6%. Among Gram negative isolates, *K. pneumoniae* (87.5%) had the highest multi drug resistance followed by *E. coli* and *P. aeruginosa* with prevalence of 83.3% and 71.4%, respectively.

**Table 4:** Antibiotic resistance profile of bacterial isolates No (%)

Isolate	AUG	CAZ	CFP	CTX	CRX	LEV	CAR	CLX	IMP	GM	AZM
*S. aureus* (11)	6(54.5)	10(90.9)	7(63.7)	6(54.5)	7(63.7)	2(18.2)	7(63.7)	8(72.7)	4(36.4)	2(18.2)	6(54.5)
CoNS (8)	1(12.5)	8(100)	2(25)	4(50)	4(50)	0	2(25)	2(25)	0	0	2(25)
*K. pneumoniae* (8)	7(87.5)	5(62.5)	5(62.5)	6(75)	7(87.5)	3(37.5)	5(62.5)	8(100)	3(37.5)	1(12.5)	3(37.5)
*P. aeuginosa* (7)	1(14.3)	6(85.7)	4(57.1)	4(57.1)	7(100)	1(14.3)	3(42.9)	6(85.7)	1(14.3)	0	1(14.3)
*Acinetobacter* species (2)	2(100)	2(100)	2(100)	2(100)	2(100)	0	0	0	0	1(50)	2(100)
*E. coli* (6)	3(50)	1(16.7)	6(100)	6(100)	6(100)	1(16.7)	4(66.7)	4(66.7)	3(50)	4(66.7)	6(100)
*Salmonella* species (4)	1(25)	1(25)	1(25)	2(50)	3(75)	0	0	3(75)	0	0	2(25)
*Shigella* species (2)	1(50)	2(100)	1(50)	0	0	0	1(50)	1(50)	0	0	1(50)

**Note:** AUG = Amoxicillin clavulanic acid; CAZ = Ceftazidime; CFP = Cefepime; CTX = Cefotaxime; CRX = Cefuroxime; LEV= Levofloxacin; CAR = Carbenicillin; CLX = Cephalexin; IMP = Imipenem; GM = Gentamicin; AZM = Azithromycin

### Detection of extended spectrum beta-lactamase (ESBL) in Gram negative isolates

**Figure 2:**
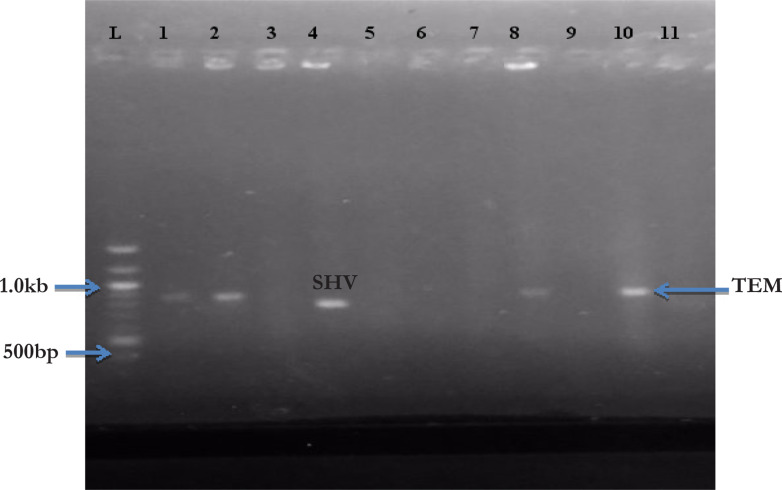
Gel image of amplified blaSHV (713 bp) and blaTEM (800 bp). L = Molecular weight marker; 1-11 = L3a, E3b, L2a, S26, L4b, M2b, A2, S5, F1b, W1b, B1

**Figure 3:**
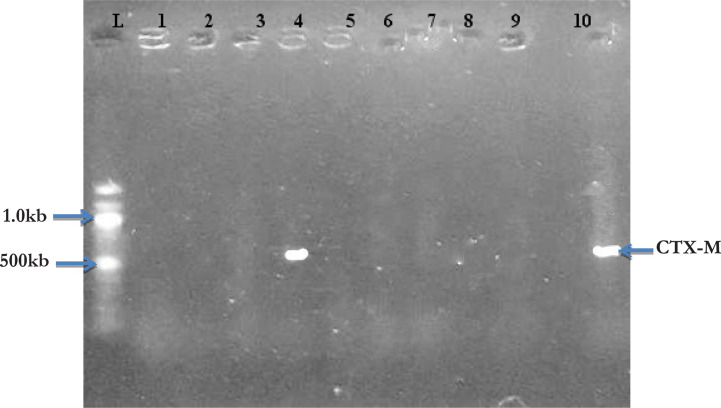
Gel image of amplified blaCTX-M (561 bp). L = Molecular weight marker; 1-10 = F1b, L2a, L2b, B4b, W1b, L2c, L4b, A2, B1 and C1b

Eight (28.5%) out of 28 Gram negative isolates were positive for extended spectrum beta-lactamase (ESBL) by phenotypic method. The isolates were: L3a (*P. aeruginosa*), E3b (*Shigella* species), B2b (*E. coli*), B4b (*E. coli*), S2b (*Acinetobacter* species), S5 (*P. aeruginosa*), P5b (*E. coli*), and C1b (*K. pneumoniae*). Figures 2 and 3 showed the amplified gel image of blaSHV, blaTEM and blaCTX-M genes. Seven (25.0%) of the Gram-negative isolates contained ESBL-encoding genes. TEM was present in 4 (14.3%) Gram negative isolates which included two *P. aeruginosa* from laboratory door and nursing station door handles, one *Shigella* species from accident and emergency section and *E. coli* from female medical ward. CTX-M were found in 2 (7.2%), one each in *E. coli* from male medical ward and *K. pneumoniae* from chief nursing officer section. SHV was found in one *Acinetobacter* species from chief nursing officer section. Two isolates that were positive phenotypically for ESBL did not contain any of the three genes while one phenotypically negative isolate carried TEM (Table 5).

**Table 5:** Molecular detection of ESBL- encoding genes in Gram negative isolates

S/N	Isolate code	Isolates	SHV	CTX-M	TEM	Phenotypic ESBL
1.	L3a	*P. aeruginosa*	-	-	+	+
2.	E3b	*Shigella* species	-	-	+	+
3.	B2b	*Escherichia coli*	-	-	-	+
4.	B4b	*Escherichia coli*	-	+	-	+
5.	S2b	*Acinetobacter* species	+	-	-	+
6.	S5	*P. aeruginosa*	-	-	+	+
7.	W1b	*Escherichia coli*	-	-	+	-
8.	C1b	*K pneumoniae*	-	+	-	+
9.	P5b	*Escherichia coli*	-	-	-	+

**Note:** + = ESBL positive; - = ESBL negative

## Discussion

Microbes are usually present in the hospital settings due to contamination of hospital surfaces by flora dissemination by patients, health professionals and people visiting the hospitals. Inanimate environmental surfaces such as hospital door handles can become directly contaminated with microorganisms after frequent exposure to healthcare givers, patients and people visiting the hospitals 22. The presence of pathogens in the hospital environment raises the risk of infection among vulnerable patients 23. Different hospitals in different countries have different microbial populations and colonization rates 24, 25. The prevalence of bacterial isolates in this study was 69%, which was lower than Bhatta et al. 25 's report of bacterial growth in 78% of samples collected. The microbiological analysis of samples from the different hospital units at Olabisi Onabanjo University in this study showed that door locations played significant role in the distribution of microorganisms. Generally, it was found that samples from maternity, laboratory, male medical ward, nursing station, virology, and female surgical ward recorded the highest bacterial load. This could be attributed to the high rates of exposure of the door handles to high-traffic users who throng in and out without washing their hands properly; spreading their flora to the door handles 26.

The bacteriological profile of frequently touched door handles showed a wide variety of Gram positive and Gram-negative organisms like S. *aureus, coagulase* negative *staphylococci, Acinetobacter* species, *P. aeruginosa, K. pneumoniae, E. coli, Salmonella* species, *Shigella* species. Bhatta et al. 25 also reported the presence of *S. aureus, E coli, Acinetobacter* species and *Pseudomonas* species from hospital settings. This study observed that *S. aureus* (22.9%) had the highest occurrence on door handles, followed by coagulase negative staphylococci (16.7%) and *K pneumoniae* (16.7%) in agreement with the work of Bhatta *et al.* 25 who also reported *S. aureus* as the most isolated bacteria from hospital door handles. Worku et al. 27 also reported *S. aureus* as the most isolated bacteria from hospital environment followed by coagulase negative staphylococci while Frank *et al.* 24 isolated *E. coli* most frequently from door handles. Contamination of door handles by *S. aureus* in a teaching hospital in Ghana was 39% in related research by Saba et al. 28, higher than the record in this study (16%).

Multidrug resistant Gram positive and Gram-negative isolates have been isolated from various hospital surfaces 25, 29, 30. *E. coli* was the most resistant isolate against imipenem. *Acinetobacter baumannii* is one organism that should be avoided in hospitals because of its persistence and ability to acquire antibiotic resistance. *A. baumannii* has appeared as a nosocomial pathogen with high levels of antibiotic resistance and resistance to conventional cleaning methods, with resistance to colistin becoming increasingly common 31. In a study comparing *P. aeruginosa* isolates from patients and hospital surfaces, Gad *et al.* 31 discovered that isolates from hospital surfaces were more likely to produce multi-resistant beta-lactamases (95%) than isolates from clinical samples (36%). *P. aeruginosa* was found to be highly resistant to ceftazidime, cefotaxime, cefepime cephalexin, and cefuroxime in this study.

Extended-spectrum beta-lactamase (ESBL)-producing Gram-negative bacteria have become an emerging global health threat and have been associated with high mortality 32 In this study, ESBL was identified phenotypically in *P. aeruginosa, Shigella* species, *E. coli, Acinetobacter* species, and *K pneumoniae* isolates from door handles. Bhatta *et al.* 25 identified 52.6% and 46.6% of *Acinetobacter* species and *E coli*, respectively as ESBL producers. Seven (25.0%) of the Gram-negative isolates encoded ESBL genes. TEM was the most detected gene, present in 14.3% Gram negative isolates which included two *P. aeruginosa* from laboratory and nursing station door handles, one *Shigella* species from accident and emergency section and *E. coli* from female medical ward. Raji *et al.* 33 reported very high prevalence of blaCTX-M type ESBL genes among the multidrug-resistant (MDR) isolates but findings from this study reported blaTEM type as the most prevalent. ESBL genes have been reported in hospital isolates from Nigeria most especially from patients and health workers 33. Touati *et al.* 5 also documented the presence of CTX-M in Enterobacteriaceae from hospital environment surfaces. However, ESBL-encoding genes are not well documented in isolates from hospitals door handles from Nigeria. The spread of ESBL genes can be rapid because most ESBL- genes are encoded on integrons and plasmids that can be transferred from one isolate to the other thus increasing the spread of antimicrobial resistance 33, 34.

Since health professionals and visitors frequently touch door handles during visits to wards, the risk of microbial contamination is extremely high 25. Door-handles were chosen for this study because of the regular and unpreventable contact with this site. Door handles are only cleaned or disinfect on a very occasional basis. The working bench, nursing stations, work surfaces, hospital floor and dressing trolley are all cleaned on a regular basis in most teaching hospitals 25 but door handles are often neglected. Transmission occurs primarily through contaminated hands, hence, the high incidence of microbial contamination found on hospital door handles, reflecting poor hand hygiene among health workers and visitors.

## Conclusions

This study revealed that door handles of different sections in hospital environment are contaminated by a variety of pathogenic microorganisms. As a result, door handles could act as fomites for the potential spread of diseases. To prevent the occurrence and dissemination of hospital acquired infections, health-care workers, patients, and visitors are encouraged to adhere to strict hand hygiene practices.
